# Use of the Professional Fulfillment Index in Pharmacists: A Confirmatory Factor Analysis

**DOI:** 10.3390/pharmacy9040158

**Published:** 2021-09-26

**Authors:** Olajide O. Fadare, William R. Doucette, Caroline A. Gaither, Jon C. Schommer, Vibhuti Arya, Brianne Bakken, David H. Kreling, David A. Mott, Matthew J. Witry

**Affiliations:** 1 College of Pharmacy, University of Iowa, Iowa City, IA 52242, USA; olajide-fadare@uiowa.edu (O.O.F.); william-doucette@uiowa.edu (W.R.D.); 2 College of Pharmacy, University of Minnesota, Minneapolis, MN 55455, USA; cgaither@umn.edu (C.A.G.); schom010@umn.edu (J.C.S.); 3 College of Pharmacy & Health Sciences, St. John’s University, Jamaica, NY 11439, USA; aryav@stjohns.edu; 4 Pharmacy School, Medical College of Wisconsin, Milwaukee, WI 53226, USA; bbakken@mcw.edu; 5 School of Pharmacy, University of Wisconsin, Madison, WI 53705, USA; david.kreling@wisc.edu (D.H.K.); david.mott@wisc.edu (D.A.M.)

**Keywords:** burnout, pharmacy, fulfillment, wellbeing, CFA

## Abstract

Most research on pharmacist professional wellbeing has focused on measuring burnout. However, using valid and reliable instruments to assess professional fulfillment in pharmacists can expand understanding of pharmacists’ professional wellbeing. This study aimed to (1) establish the validity of the Professional Fulfillment Index (PFI) for a sample of pharmacists licensed in the United States (US) using confirmatory factor analysis (CFA), and (2) compare the professional wellbeing of pharmacists across demographics and work settings. Data for this study were obtained from the 2019 National Pharmacy Workforce Survey (NPWS). The survey assessed pharmacist professional wellbeing using the PFI. The model fit of the PFI was assessed using CFA. Multiple linear regression was used to compare pharmacist wellbeing across demographics and work settings. The CFA affirmed that the PFI possesses a satisfactory model fit for use in pharmacists. Regression analysis showed higher burnout (work exhaustion and interpersonal disengagement) was associated with decreasing age, being female, working more hours, and working in a community pharmacy. Higher professional fulfillment was associated with men, and working in non-community pharmacy work settings. The PFI is a psychometrically reliable and valid instrument for assessing the professional wellbeing of pharmacists.

## 1. Introduction

Pharmacists and other healthcare professionals have been experiencing high levels of exhaustion, overwork, and time pressures. These conditions are attracting attention from stakeholders and policymakers in healthcare because of the potential negative effects on the workforce, their organizations, and the patients they serve [1,2,3]. Burnout is defined as a state of physical, emotional, and mental exhaustion that results from long-term involvement with psychologically demanding work situations [4,5]. Burnout among health professionals can lead to job dissatisfaction, increased intention to leave, reduced quality of care, and poorer health outcomes for patients [3,6]. Much of the literature on professional wellbeing focuses on measuring burnout. However, there is growing recognition that lowering burnout does not necessarily translate to more professional fulfillment [7,8]. Professional fulfillment is defined as a sense of engagement, reward, and contentment with one’s career [9]. Increased recognition of professional fulfillment as a critical aspect of pharmacist wellbeing expands the scope of research and evaluation efforts to include the design, testing, and implementation of interventions for improving professional fulfillment in addition to efforts to reduce burnout. Hence, there is a need for valid measurement models and instruments for measuring professional fulfillment alongside burnout.

Most measurement instruments in the domain of professional wellbeing focus on assessing burnout. The most widely used measure is the Maslach Burnout Inventory (MBI) [10]. The MBI assesses burnout on three dimensions of emotional exhaustion, depersonalization, and personal accomplishment. The personal accomplishment scale of the MBI assesses perceived reduction in professional efficacy (feelings of low competence) as a positive-valence component of burnout [11,12]. However, this conceptualization does not capture many of the intrinsic characteristics of work that contribute to professional fulfillment [9,13]. Another measure, the Copenhagen Burnout Inventory (CBI) was developed to address some of the limitations of the MBI [14]; however, this three-domain measure focuses only on burnout, with no assessment of fulfillment or accomplishment [15]. This exclusive focus of measurement instruments on burnout may not capture the whole experience of pharmacy professional’s wellbeing, as they primarily emphasize the negative aspects of a pharmacist’s work experience. Other aspects of work, such as task control, engagement, meaningfulness, positive emotional affect (happiness and enthusiasm), and a sense of contribution have been found to predict satisfaction, work engagement, and positive professional outlook in health professionals [9,16,17].

The Professional Fulfillment Index (PFI) was developed to capture both burnout and professional fulfillment in the evaluation and assessment of professional wellbeing [18]. The PFI is a 16-item index that measures burnout and professional fulfillment. Burnout is measured using two scales that represent two dimensions of work exhaustion and interpersonal disengagement, respectively. The PFI also includes a scale to measure professional fulfillment. The Professional Fulfillment (PF) scale “assesses the degree of intrinsic positive rewards the individual derives from his or her work”; the Work Exhaustion (WE) scale “assesses symptoms of exhaustion”, and the Interpersonal Disengagement (ID) scale “assesses empathy and connectedness with others” [18].

In addition to the inclusion of a professional fulfillment scale, the PFI is designed to assess respondents’ perceived burnout and professional fulfillment over the 2 weeks before taking the survey. The inclusion of a time-cap is a distinct feature of the PFI that is absent in most other burnout measures. The PFI authors posit that including a time boundary simplifies the interpretation of changes in item scores across time points, thereby enabling evaluators and researchers to measure current wellbeing levels and assess the impact of short-term interventions [18]. While the performance of the PFI has been established in a study of physicians, its validity for use in pharmacy professionals is yet to be determined.

## 2. Objectives

The objectives of these analyses were to (1) establish the validity of the PFI for a sample of US pharmacists using confirmatory factor analysis (CFA), and (2) compare the professional wellbeing of pharmacists across pharmacists’ demographics.

## 3. Methods

### 3.1. Data Collection

This analysis used data collected as part of the National Pharmacy Workforce Survey, which was conducted in 2019. A description of the electronic survey methods and assessment of nonresponse bias and potential limitations are reported elsewhere [19]. The survey data were examined for usable responses. A response is considered usable when values for age, gender, work setting, employment status, and hours worked per week are present.

### 3.2. Descriptive Statistics

The survey data were assessed for missingness using the guideline recommended by Mirzaei and colleagues [20]. The data were assessed for normality visually by examining boxplots and histograms of score distributions for each variable in the dataset, and by using Mardia’s test of multivariate normality. The presence of multivariate outliers was assessed using the minimum covariance determinant based on a robust Mahanalobi’s distance. The distribution of pharmacists across age categories, gender, and work setting was computed. The average scores for professional fulfillment, work exhaustion, and interpersonal disengagement for each demographic group were determined and compared using independent sample t-tests and one-way ANOVA. The correlations between PFI items and pharmacist age and hours worked per week were also computed, and the internal consistency reliability of each of the scales was assessed using Cronbach’s alpha and inter-item correlation.

### 3.3. Measures

The PFI is a 16-item measure that assesses two aspects of pharmacist professional wellbeing [18]. These are professional fulfillment and burnout. Professional fulfillment (PF) was measured using a 6-item, 5-point scale scored from 0 (not at all true) to 4 (completely true). An example item on the PF scale is “during the past two weeks my work is satisfying to me.” Burnout was assessed on two dimensions: work exhaustion (WE) and interpersonal disengagement (ID). WE was measured using a 4-item scale. An example item on this scale is “during the past two weeks I have felt physically exhausted at work.” The original ID scale comprised six items, with three items assessing pharmacists’ relationships with professional colleagues and three items assessing relationships with patients. However, the three items on the original ID scale assessing pharmacist relationship with patients were excluded from this analysis because the survey was designed to restrict those three items to only a portion of the sample based on the belief that it was not valid to ask pharmacists who do not regularly interact with patients (e.g., hospital administrators, some hospital pharmacists, and industry or non-clinical academic researchers) to respond to these items. While excluding these three items may not be ideal, this dimension is conceptualized to measure a single latent variable, and thus we chose to proceed with the 3 other items to represent the domain. Therefore, ID was measured using a reduced 3-item scale. An example item on this scale is “during the past two weeks I have felt less connected with my colleagues.” Both WE and ID scales are scored from 0 (not at all) to 4 (extremely).

The workforce survey also assessed pharmacist demographics, work setting, and work characteristics, as well as other topics, as reported elsewhere [19]. For this study, we grouped the sample into five work settings. These are: (1) community pharmacists (comprising independent, small chain, large chain, mass merchandiser, supermarket, and health system retail pharmacies), (2) physician office/outpatient pharmacists, (3) institutionalized care pharmacists (comprising hospital and health systems), (4) noninstitutionalized care pharmacists (comprising mail order, managed care, pharmacy benefits associations, nursing home, home health/infusion, and specialty pharmacy), and (5) other roles (comprising academia, industry, specialty pharmacy, information technology, and others).

### 3.4. Confirmatory Factor Analysis (CFA)

Confirmatory factor analysis (CFA) was used to confirm the fit of the 3-factor structure of the PFI in the study sample. Considering that large sample sizes may lead to excess power for the CFA, which could increase Type (I) error, cross-validation was applied [21]. In this process, the sample (without outliers) was randomly split into two halves—sample A (*n* =2296) and sample B (*n* = 2295). Sample A was used as the test sample to test the hypothesized model, and sample B was used as the validation sample. CFA model fit was evaluated using the following fit indices: model chi-square (χ^2^), standardized root mean square residual (SRMR), Tucker–Lewis index (TLI), comparative fit index (CFI), and root mean square error of approximation (RMSEA). A cut-point of 0.05 was set for SRMR, with smaller values considered as an indication of satisfactory model fit [22]. Perry et al. (2015) suggested that traditional cut-points of >0.95 for TLI and CFI for estimating goodness of fit may be too stringent when a scale representing multiple latent factors is modeled in a diverse sample [23]. Therefore, a cut-point of >0.90 for TLI and CFI was considered satisfactory for estimating goodness of fit in the CFA. In the CFA, the three latent factors (professional fulfillment, work exhaustion, and interpersonal disengagement) were allowed to correlate and were scaled by using one of the items on each variable as a reference indicator for the respective variable. Additionally, each PFI item was constrained to load on only one factor at a time. The average variance extracted (AVE) for each construct and the shared variance between constructs were compared to establish discriminant validity between constructs.

### 3.5. Measurement Invariance (Multi-Group Analysis)

Multi-group analysis was used to assess whether the PFI performs consistently across pharmacist work settings. In this analysis, model fit for each group was first assessed individually to determine suitability for inclusion in the multi-group analysis. Next, multi-group analysis was conducted, in which constraints of equal factor structure, equal factor loadings, and equal intercept across groups were imposed on the model. The following criteria were used to evaluate the fit of measurement invariance models: ∆CFI < 0.002, ∆RMSEA < 0.015, ∆SRMR < 0.030. Less focus was placed on ∆χ^2^ due to its excessive sensitivity to small and unimportant deviations from a perfect model in large samples [24,25,26]. 

### 3.6. Regression Analysis

Pharmacists’ professional fulfillment and burnout (work exhaustion and interpersonal disengagement) were compared across age groups and practice types using one-way analysis of variance (ANOVA). Multiple linear regression was used to assess the relationship between pharmacist demographics and work setting (predictors), and professional fulfillment, work exhaustion, and interpersonal disengagement as outcome variables. Cohen’s effect size criteria (0.01 ≤ R^2^ < 0.09 = small; 0.09 ≤ R^2^ < 0.25 = medium; R^2^ ≥ 0.25 = large) were used for evaluating the overall strength of relationship between the explanatory variables and the outcome variables in the regression models [27]. All statistical analyses were performed using R v4.0.5 at an a priori significance level of 0.05.

## 4. Results

### 4.1. Descriptive Statistics

A random sample of 96,000 licensed US pharmacists was sent the NPWS survey electronically via email. In total, 8456 survey recipients clicked the survey link to open the survey; 4716 usable responses were received. This gave a usable response rate of 55.6%, using the number of opened surveys as the denominator. The percentage missingness in the usable response dataset (*n* = 4716) was 62.5%. Since this percentage was greater than 40%, the dataset was investigated for missingness and was determined to be missing at random (MAR). As this was a large survey sent to a large sample of pharmacists diverse in their practice setting, it can be expected that respondents responded to items on the survey based on how relevant items are to their professional pharmacy practice. Most of the missingness in the dataset was observed among variables related to the uniqueness of pharmacists’ practice type (e.g., opioid dispensing, residency training requirement, preceptorship). However, among variables included in these analyses, the PFI items age, and hours worked per week had 0% missingness. Gender had less than 0.01% missingness, which was due to how responses were coded for analysis. Eleven gender responses were recoded as “NA” due to the low representativeness of this group in the overall sample, which would not allow for reliable estimates.

The mean age for the study sample was 44 years (SD = 13.1), ranging from 20 to 87 years. In total, 64.8% identified as women, and 94.2% were practicing pharmacists at the time the survey was conducted. Mean professional fulfillment, work exhaustion, and interpersonal disengagement for each demographic group and work setting were computed, as shown in Table 1.

The Mardia’s test for multivariate normality was significant (*p* < 0.001), suggesting a non-normal distribution of responses across the PFI items. Examining the data showed some items with skewness and kurtosis > |1| [28] (Table 2).

In total, 125 multivariate outliers were identified. The scores of these outliers had extreme values and, as they were not due to missingness, they were considered to represent extreme cases in the sample. These outliers were excluded from subsequent analyses. To accommodate non-normality and the ordinal nature of the variables, the diagonally weighted least square (WLSMV) estimator was used in the CFA [29,30]. The three scales on the PFI showed high internal consistency reliability with Cronbach’s alpha of α_PF_ = 0.92, α_WE_ = 0.92, and α_ID_ = 0.92 for each scale, respectively. The average inter-item correlations were PF = 0.67, WE = 0.73, and ID = 0.78. Correlations between PFI items, pharmacists’ age and hours worked per week are shown in Table 3.

### 4.2. Confirmatory Factor Analysis (CFA)

Using sample A (*n* = 2296), the CFA model had satisfactory goodness-of-fit on most fit indices (χ^2^_(62)_ = 1069.99, *p* < 0.001, CFI = 0.997, TLI = 0.997, SRMR = 0.042, RMSEA = 0.084 (0.080, 0.089)). As shown in Table 4, all standardized factor loadings were large (range = 0.76 to 0.95) and statistically significant (*p* < 0.05), indicating that scale items on the PFI are of high measurement quality. Item-error variances were also significant (*p* < 0.05). Although the chi-square (χ^2^) statistic is upwardly biased in large samples with non-normality, and RMSEA is usually biased upwards in models with a small number of high-quality indicators (factor loadings > 0.80) and a low degree of freedom (df), retaining the specified model was based on inclusion and consideration of fit indices that are more robust against the effect of sample size and df in model evaluation [31,32].

For measurement invariance testing, sample A was divided into five subgroups based on pharmacists’ work setting. When individually assessed for suitability of inclusion in measurement invariance analysis, acceptable model fit was obtained for each of the five groups. Hence, all five groups were included in the measurement invariance analysis. Three levels of invariance across pharmacists’ work setting were tested. These were configural invariance (test of equality of factor structure), metric invariance (test of equality of factor loadings), and scalar invariance (test of equality of intercepts). Satisfactory fits were obtained for the measurement invariance models. The fit indices were as follows for (1) configural invariance—χ^2^_(310)_ = 1233.68, *p* < 0.001, CFI = 0.997, TLI = 0.997, SRMR = 0.049, RMSEA = 0.081 (0.076, 0.085); (2) metric invariance—χ^2^_(350)_ = 1520.60, *p* < 0.001, CFI = 0.997, TLI = 0.996, SRMR = 0.052, RMSEA = 0.085 (0.081, 0.090); and (3) scalar invariance—χ^2^_(494)_ = 1559.37, *p* < 0.001, CFI = 0.997, TLI = 0.998, SRMR = 0.049, RMSEA = 0.069 (0.065, 0.072). The imposition of successive equality constraints for configural, metric, and scalar invariance did not significantly degrade the fit of the model (∆CFI < 0.002, ∆RMSEA < 0.015, ∆SRMR < 0.030), which indicates that the measurement of professional fulfillment, work exhaustion, and interpersonal disengagement by the PFI is invariant across pharmacists’ work settings. 

**Figure 1:**
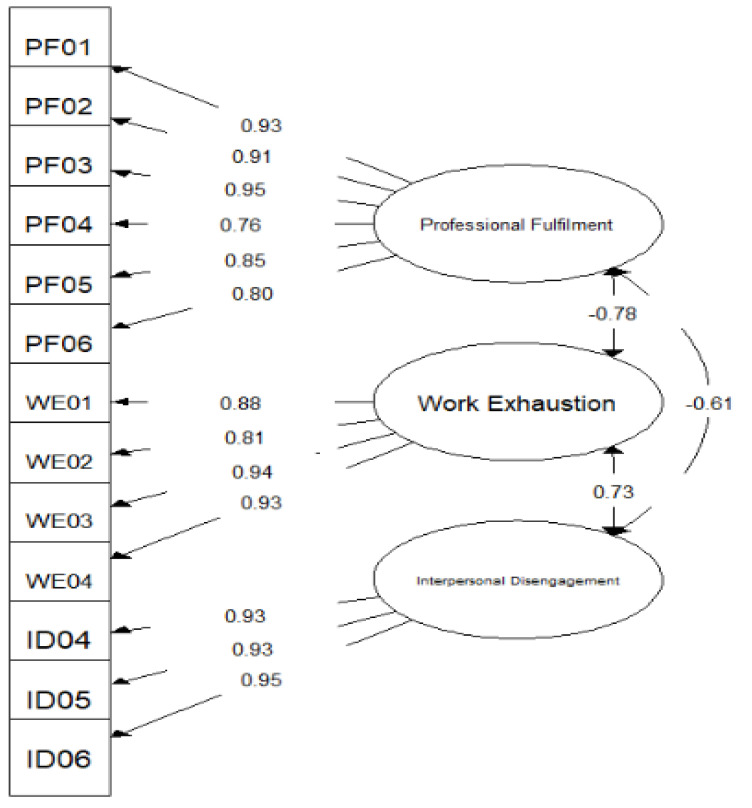
Path diagram for CFA Model. χ^2^_(62)_ = 1069.999, *p* < 0.001, CFI = 0.997, TLI = 0.997, SRMR = 0.042, RMSEA = 0.084 (CI: 0.080; 0.089).

Cross-validating these analyses using the validation sample (sample B, *n* = 2295) confirmed measurement invariance across pharmacists’ work setting (∆CFI < 0.002, ∆RMSEA < 0.015, ∆SRMR < 0.030). The inter-factor correlations obtained from the model (Figure 1) were −0.61 for the relationship between PF and ID, −0.78 for the relationship between PF and WE, and 0.73 for the relationship between WE and ID. The shared variance between PF and ID was 0.37, PF and WE was 0.61, and WE and ID was 0.53. The average variance extracted (AVE) for PF was 0.75, WE was 0.79, and ID was 0.87. Since the average variance extracted for each construct was greater than its shared variance with any other construct, discriminant validity is supported [33].

### 4.3. Comparison of Professional Wellbeing across Pharmacist Demographics and Work Setting

There was a significant difference in pharmacists’ professional fulfillment, work exhaustion, and interpersonal disengagement across pharmacists’ work setting (Table 1). Regression analysis (Table 5) showed that age was negatively associated with burnout, while the number of hours worked per week was positively associated with burnout. Male pharmacists reported less burnout than female pharmacists, and community pharmacists reported higher levels of burnout than pharmacists in other work settings. Age was not significantly associated with professional fulfillment but community pharmacists reported lower perceived professional fulfillment compared to pharmacists in other work settings, and the number of hours worked per week was negatively associated with professional fulfillment. The demographic variables included in the regression models had small effect sizes (R^2^ < 0.06) in explaining professional fulfillment and interpersonal disengagement in pharmacists, and a medium effect (R^2^ = 0.19) in explaining work exhaustion in pharmacists.

## 5. Discussion

The study findings show that the three PFI measures have high internal consistency and reliability for use in the assessment of professional fulfillment and burnout in a sample of US pharmacists. Additionally, the hypothesized three-factor model presented by the PFI authors fits this sample of pharmacists adequately with strong discriminant validity among the three latent factors [28]. Consistent with the theoretical basis of the PFI, each subscale of the PFI can be used independently to assess various dimensions of pharmacists’ professional wellbeing. However, while scores on the work exhaustion and interpersonal disengagement scales can be combined to assess burnout, the professional fulfillment scores do not necessarily represent the direct opposite of work exhaustion and interpersonal disengagement for pharmacists, as the absence of these burnout dimensions does not always imply professional fulfillment [7,8,18]. Hence, scores from the three scales should not be combined to create a single index score. The PFI provides a tool to measure professional fulfillment in pharmacists, which we consider to be an important contribution to shifting the focus of current discussions about pharmacist professional wellbeing from trying to reduce burnout to adding consideration of professional fulfillment [34]. 

### Assessing Pharmacists’ Professional Wellbeing across Pharmacists’ Work Setting

Another objective of this study was to assess whether the PFI maintains its psychometric properties across pharmacists’ work settings. The rationale for this is that pharmacists may differ in their perceptions and experiences of burnout (work exhaustion and interpersonal disengagement) and professional fulfillment depending on their work setting. For instance, pharmacists who have a lot of patient contacts may experience burnout differently compared to pharmacists who have little or no patient contact, but more of other types of work activities [2]. The practical implications of the observed measurement invariance for pharmacy practice evaluation and research using the PFI include that the PFI is a consistent measure of professional fulfillment, work exhaustion, and interpersonal disengagement across pharmacist work settings. Thus, PFI measurements obtained from a heterogeneous population of pharmacists can be used in comparative statistical analysis, and yield meaningful and interpretable results [22]. This invariance property positions the PFI as a measurement tool that pharmacy organizations, policy planners, and administrators can use to compare burnout and professional fulfillment in their pharmacists, and to target efforts to improve pharmacists’ wellbeing.

Moreover, significant differences in the professional wellbeing of pharmacists across work settings suggest the job characteristics of pharmacists’ areas of practice impact their experience of professional fulfillment and burnout [35]. For instance, pharmacists at community pharmacies reported the lowest average professional fulfillment and highest average work exhaustion and interpersonal disengagement compared to pharmacists in other work settings. This could be due to the increased demand for dispensing and prescription management services, such that community pharmacists are taking less time than they desire to provide more patient care [36,37]. While this comparison suggests an inverse relationship between professional fulfillment and burnout, other research suggests that the absence of burnout does not necessarily lead directly to professional fulfillment [7]. Unmeasured variables in this analysis, such as workload, task variety, and job control, could explain why community pharmacists reported lower professional fulfillment and higher burnout than their colleagues in other work settings [38].

The regression analysis also showed that burnout (work exhaustion and interpersonal disengagement) decreases with increasing age; that is, older pharmacists experience these dimensions of burnout less than younger pharmacists. One potential explanation could be that older pharmacists have developed positive coping techniques, acquired from experiences when faced with a stressful situation [39,40]. While work stress might be buffered by individual pharmacist’s coping skills, this is not sufficient to engender professional fulfillment for pharmacists without consideration of other aspects of work [41]. There was no significant difference in reported professional fulfillment based on age.

Additionally, pharmacists who are men reported lower burnout (work exhaustion and interpersonal disengagement) than pharmacists who are women. The evidence on the effect of gender on burnout is mixed, as some studies showed no statistically significant association, while some studies showed a statistically significant association between burnout and gender among pharmacists and other healthcare professionals [42,43]. Some studies have also shown statistically significantly higher burnout among male pharmacists compared to female pharmacists [44,45]. This inconsistency in findings suggests that further research is needed to elucidate how gender influences burnout in pharmacists. Additionally, the small and medium effects of age, gender, and work setting in explaining pharmacists’ professional fulfillment and burnout respectively suggest the potential salience of other factors, such as job demands, job resources, and workplace characteristics, in predicting pharmacists’ professional wellbeing. These are important because of the immutability of gender and age. Further research will be needed on how the COVID-19 pandemic acutely affected work-life among pharmacists.

## 6. Implications for Pharmacy Practice

The PFI is a psychometrically valid and reliable instrument applicable to diverse work settings, which pharmacy managers, administrators, and perhaps policymakers can use to assess the professional wellbeing of pharmacists. We posit that being able to assess various dimensions of burnout using the PFI can provide pharmacy managers with valuable insight on which aspects of work should receive more focus for both short- and long-term interventions. For instance, our study findings show a greater association between pharmacist demographic characteristics and work exhaustion compared to interpersonal disengagement. Pharmacy organizations seeking to reduce burnout in their pharmacists may achieve results, at least in the short term, by focusing on indicators of work exhaustion. Additionally, with the PFI, pharmacy organizations can identify indicators to be targeted for interventions to raise pharmacist’s professional fulfillment. This is a salient addition of the PFI to the discourse about the professional wellbeing of pharmacists, based on recognizing that the absence of burnout does not imply that pharmacists find their jobs fulfilling.

## 7. Limitations

The findings of this study should be interpreted within the context of study limitations. Although the authors of the PFI conceptualized the PFI to measure respondents’ perceived professional fulfillment and burnout over the previous two weeks, the PFI was used cross-sectionally. Hence, measurement invariance in the PFI across time could not be assessed. Additionally, we had a large sample for this national survey, and some fit statistics are sensitive to sample size in a multi-group CFA framework. It is possible that differences in group size could bias the fit indices obtained from the measurement invariance tests. To address this, we used multiple fit indices, including those that are not influenced by sample size, for these assessments. The survey response rate was low, at 55.8%, which increases the potential for nonresponse bias. Witry et al. (2020) provided a full description of how 2019 NPWS survey respondents differ from the population of licensed pharmacists in the United States [19].

## 8. Future Research

Studies that measure professional fulfillment and burnout longitudinally are needed to demonstrate and validate the PFI’s sensitivity to changes in professional fulfillment and burnout across time, as proposed by the developers. An empirical assessment of time-related change in professional fulfillment will be an important contribution to pharmacy practice research, as these longitudinal studies may enable researchers to capture the influence of other personality- and non-work-related factors on pharmacists’ burnout and professional fulfillment. More research is needed to investigate how different aspects of pharmacists’ work-life influence professional wellbeing, as this may help administrators to plan and properly target interventions aimed at improving pharmacists’ professional wellbeing. Additionally, future research should investigate how and why older pharmacists experience lower levels of burnout, with the possibility of using such insights to develop interventions for younger pharmacists and those working in community settings to boost their resilience against burnout and instill greater fulfillment. Considerations of part-time or full-time employment should be included in future studies assessing the relationship between work hours and pharmacists’ professional wellbeing. Future research focused on community pharmacy should consider the characteristics of different community pharmacy work settings (e.g., small-chain, independent, supermarket pharmacies), such as ownership structure, prescription volume, service mix, job demands, job resources, and others.

## 9. Conclusions

This study provides evidence for the validity and reliability of the PFI as a measurement tool for assessing the professional wellbeing of pharmacists across different work settings. Age appears to be protective against work exhaustion and interpersonal disengagement; hence pharmacy managers can explore ways of leveraging the experience of older pharmacists in their employ in their efforts to reduce pharmacist burnout. Additionally, the peculiarities of different work settings should be considered when designing interventions to improve the professional wellbeing of pharmacists.

## Figures and Tables

**Table 1:** Sample distribution and average professional wellbeing for pharmacists (*n* = 4716).

Characteristic	*n* (%)	PF (Professional Fulfillment)	WE (Work Exhaustion)	ID (Interpersonal Disengagement)
Age (years)		Mean (SD)	Mean (SD)	Mean (SD)
≤30	810 (17.2)	3.06 (0.97)	2.98 (1.06)	2.38 (1.00)
31–40	1376 (29.2)	3.01 (0.98)	2.97 (1.08)	2.32 (0.99)
41–50	858 (18.2)	2.94 (1.02)	2.96 (1.14)	2.34 (1.04)
51–60	1039 (22.0)	3.02 (0.99)	2.74 (1.07)	2.17 (0.95)
>60	632 (13.4)	3.28 (1.02)	2.35 (1.08)	1.92 (0.89)
		*p =* 0.2655	* p < 0.001	* p < 0.001
Gender				
Male	1646 (34.9)	3.10 (1.04)	2.69 (1.11)	2.20 (1.01)
Female	3059 (64.8)	3.02 (0.98)	2.92 (1.09)	2.28 (0.98)
Non-binary	11 (0.2)	-	-	-
		* p = 0.0045	* p < 0.001	*p* = 0.075
Work Setting for Pharmacists				
Community	2162 (45.84)	2.79 (0.98)	3.21 (1.10)	2.41 (1.00)
Institutionalized care	1220 (25.87)	3.24 (0.92)	2.58 (0.97)	2.16 (0.97)
Physician office/Outpatient	259 (5.49)	3.34 (0.95)	2.53 (1.00)	2.20 (0.95)
Noninstitutionalized care	572 (12.13)	3.09 (1.03)	2.54 (1.07)	2.10 (0.97)
Other roles	503 (12.01)	3.48 (0.97)	2.34 (0.98)	1.94 (0.90)
		* p < 0.001	* p < 0.001	* p < 0.001

Note. Community pharmacists (comprise independent, small chain, large chain, mass merchandiser, supermarket, and health system retail pharmacies); Physician office/outpatient pharmacists; Institutionalized care pharmacists (comprise hospital and health systems); Noninstitutionalized care pharmacists (comprise mail order, managed care, pharmacy benefits associations, nursing home, home health/infusion, and specialty pharmacy); Other roles (comprise academia, industry, specialty pharmacy, information technology, and others). * *p* < 0.05 indicates a statistically significant difference in group means.

**Table 2:** Descriptive statistics of PFI items and pharmacists’ demographics (*n* = 4716).

^3^ PFI Item	Mean	SD	Median	Skew	Kurtosis	IIC
PF01	2.77	1.16	3	0.109	−0.859	0.68
PF02	3.08	1.20	3	−0.152	−0.943	0.70
PF03	3.01	1.20	3	−0.071	−0.955	0.73
PF04	2.91	1.11	3	−0.012	−0.757	0.57
PF05	3.40	1.15	4	−0.386	−0.738	0.67
PF06	3.11	1.24	3	−0.11	−1.011	0.64
WE01	2.77	1.23	3	0.271	−0.844	0.73
WE02	2.93	1.24	3	0.103	−0.953	0.71
WE03	2.75	1.19	3	0.286	−0.739	0.72
WE04	2.89	1.28	3	0.167	−1.021	0.77
ID04	2.18	1.12	2	0.663	−0.334	0.80
ID05	2.11	1.09	2	0.777	−0.084	0.78
ID06	2.17	1.13	2	0.732	−0.245	0.79
Age	44.39	13.10	-	0.284	−0.924	-
HWPW	40.64	11.37	-	−0.311	2.456	-
Work setting	-	-	-	−0.005	−0.811	-

Note. PF = professional fulfillment, WE = work exhaustion, ID = interpersonal disengagement, HWPW = hours worked per week, IIC = inter-item correlation. ^3^: The PFI items were measured on a 5-point scale (0 = not at all true to 4 = completely true) and are defined as follows. During the past two weeks, I… PF01 = I feel happy at work, PF02 = I feel worthwhile at work, PF03 = My works is satisfying to me, PF04 = I feel control when dealing with difficult problems at work, PF05 = My work is meaningful to me, PF06 = I’m contributing professionally in the ways I value most; During the past two weeks I have felt… WE01 = A sense of dread when I think about work I have to do, WE02 = Physically exhausted at work, WE03 = Lacking in enthusiasm at work, WE04 = Emotionally exhausted at work; During the past two weeks my job has contributed to me feeling… ID04 = Less empathetic with my colleagues, ID05 = Less sensitive to others’ feelings/emotions, ID06 = Less connected with my colleagues.

**Table 3:** Correlations between PFI items and pharmacists’ demographic variables.

	PF01	PF02	PF03	PF04	PF05	PF06	WE01	WE02	WE03	WE04	ID04	ID05	ID06	Age
**PF01**														
**PF02**	0.77													
**PF03**	0.79	0.81												
**PF04**	0.60	0.61	0.60											
**PF05**	0.63	0.67	0.76	0.57										
**PF06**	0.61	0.63	0.70	0.55	0.71									
**WE01**	−0.65	−0.54	−0.58	−0.47	−0.44	−0.44								
**WE02**	−0.56	−0.46	−0.48	−0.39	−0.34	−0.36	0.71							
**WE03**	−0.70	−0.64	−0.67	−0.51	−0.56	−0.54	0.74	0.64						
**WE04**	−0.67	−0.56	−0.58	−0.49	−0.44	−0.45	0.76	0.78	0.78					
**ID04**	−0.46	−0.42	−0.41	−0.38	−0.35	−0.36	0.49	0.43	0.55	0.54				
**ID05**	−0.48	−0.44	−0.45	−0.38	−0.41	−0.41	0.53	0.47	0.59	0.58	0.79			
**ID06**	−0.51	−0.47	−0.45	−0.42	−0.38	−0.40	0.51	0.46	0.57	0.57	0.81	0.76		
**Age**	0.05	0.06	0.05	0.04	0.02	0.06	−0.14	−0.14	−0.17	−0.17	−0.12	−0.15	−0.09	
**HWPW**	−0.10	−0.06	−0.05	−0.06	−0.01	0.01	0.19	0.17	0.14	0.19	0.13	0.15	0.12	−0.17

**Table 4:** Standardized factor loadings from CFA model (sample A, *n* = 2296).

PFI Item	λ	95% CI	Error Variance
PF01	0.934 ***	(0.926; 0.942)	0.128 ***
PF02	0.905 ***	(0.896; 0.914)	0.181 ***
PF03	0.951 ***	(0.941; 0.954)	0.100 ***
PF04	0.760 ***	(0.742; 0.779)	0.422 ***
PF05	0.847 ***	(0.833; 0.858)	0.285 ***
PF06	0.800 ***	(0.783; 0.816)	0.361 ***
WE01	0.876 ***	(0.863; 0.889)	0.232 ***
WE02	0.809 ***	(0.793; 0.825)	0.346 ***
WE03	0.937 ***	(0.928; 0.945)	0.123 ***
WE04	0.931 ***	(0.922; 0.940)	0.133 ***
ID04	0.934 ***	(0.959; 0.942)	0.128 ***
ID05	0.929 ***	(0.920; 0.938)	0.136 ***
ID06	0.952 ***	(0.945; 0.960)	0.093 ***

Note: λ = factor loadings. *** *p* < 0.001 for all factor loadings and error variances.

**Table 5:** Regression coefficients comparing pharmacist demographics with pharmacist wellbeing outcomes (*n* = 4580).

Variables	Professional Fulfillment (β)	Work Exhaustion (β)	Interpersonal Disengagement (β)
Age	0.017	−0.088 ***	−0.−069 ***
^1^ Gender (male)	0.105 ***	−0.245 ***	−0.078 **
^2^ Institutionalized care pharmacist	0.462 ***	−0.646 ***	−0.267 ***
Noninstitutional care pharmacist	0.324 ***	−0.673 ***	−0.319 ***
Physician office/Outpatient	0.582 ***	−0.730 ***	−0.254 ***
Other roles	0.738 ***	−0.920 ***	−0.509 ***
Hours worked per week (HWPW)	−0.125 ***	0.364 ***	0.259 ***
Adjusted R-squared	0.07811	0.183	0.0755
F	56.42 *	146.6 *	54.46 *

Note: ^1^: reference group = female; ^2^: reference group for work setting = community pharmacists; Burnout variable is a composite of work exhaustion and interpersonal disengagement dimensions of the PFI community pharmacists (comprising independent, small chain, large chain, mass merchandiser, supermarket, and health system retail pharmacies); Physician office/Outpatient pharmacists; Institutionalized care pharmacists (comprise hospital and health systems); Noninstitutionalized care pharmacists (comprise mail order, managed care, pharmacy benefits associations, nursing home, home health/infusion, and specialty pharmacy); Other roles (comprise academia, industry, specialty pharmacy, information technology, and others). *β* = regression coefficient. *** *p* < 0.001, ** *p* < 0.01, * *p* < 0.05.
